# Erratum

**DOI:** 10.1093/ijnp/pyx006

**Published:** 2017-02-24

Potkin, SG, Loze, J-Y, Forray C, Baker RA, Sapin C, Peters-Strickland T, Beillat M, Nylander A-G, Hertel P, Nitchsky Schmidt S, Eramo A, Hansen K, Naber D (2016) Multidimensional assessment of functional outcomes in schizophrenia: results from QUALIFY, a head-to-head trial of aripiprazole once-monthly and paliperidone palmitate. Int J Neuropsychopharmacol. 20: 40–49.

In “Multidimensional Assessment of Functional Outcomes in Schizophrenia: Results From QUALIFY, a Head-to-Head Trial of Aripiprazole Once-Monthly and Paliperidone Palmitate,” *The International Journal of Neuropsychopharmacology*, DOI: 10.1093/ijnp/pyw093, the Significance Statement included errors. The correct Significance Statement is below. This has been corrected in the original article.

**Significance Statement**

The QUALIFY study (QUAlity of LIfe with AbiliFY Maintena) compared aripiprazole once-monthly 400 mg with paliperidone palmitate across multifaceted clinical and functional scales addressing changes in symptoms, quality of life, and functional capacity. Overall, our findings show consistently greater improvements with aripiprazole once-monthly 400 mg vs paliperidone palmitate across scales reflecting a broad range of symptoms and functioning, regardless of whether the raters were blinded or not and whether the scale was rated by the patient or clinician. These results across multiple domains of health-related quality of life and functioning and in enhanced work readiness help to differentiate aripiprazole once-monthly 400 mg and paliperidone palmitate, 2 long-acting injectable antipsychotics with different pharmacologic profiles. Aripiprazole is a partial agonist at dopamine D_2_ and serotonin 5HT_1A_ receptors and an antagonist at 5HT_2A_ receptors while paliperidone is an antagonist at D_2_ and 5HT_2A_ receptors. Our results may help provide guidance to clinicians when choosing between antipsychotic treatments and psychosocial options for their patients with schizophrenia.

